# An exploratory analysis of obsessive-compulsive personality traits, pathologic anger and quality of life among trauma-exposed veterans

**DOI:** 10.1016/j.comppsych.2025.152628

**Published:** 2025-08-20

**Authors:** Meghan J. Kulak, Meghan A. Gonsalves, Timothy Y. Mariano, Anthony Pinto, Benjamin D. Greenberg, Jennifer Barredo, Zachary Kunicki, M. Tracie Shea

**Affiliations:** a Department of Psychiatry and Human Behavior, Alpert Medical School of Brown University, Providence, RI, USA; b Center for Neurorestoration and Neurotechnology, Providence VA Healthcare System, Providence, RI, USA; c Northwell Health OCD Center, Glen Oaks, NY, USA

**Keywords:** Post-traumatic stress disorder, PTSD, Combat trauma, Obsessive-compulsive personality disorder, OCPD, Anger, Quality of life

## Abstract

**Introduction:**

Trauma exposure and post-traumatic stress disorder (PTSD) are associated with high rates of co-occurring personality pathology, including Obsessive Compulsive Personality Disorder (OCPD). OCPD is characterized by rigidity and perfectionism, leading to internalized distress, interpersonal difficulties, and lower quality of life (QOL). Individuals with OCPD often report high levels of anger, which may exacerbate distress and interpersonal issues. However, the relationship between OCPD, anger, and QOL in trauma-exposed Veterans remains understudied.

**Materials and methods:**

92 Veterans with warzone trauma, hyperarousal symptoms, and moderate to severe problems with anger were recruited from a VA Medical Center for a study on cognitive-behavioral therapy for anger. Assessments included standardized interviews on demographics and trauma history, as well as self-reports on OCPD and borderline personality disorder (BPD) traits, anger, and QOL.

**Results:**

OCPD scores were significantly correlated with higher state anger (*r* = 0.276, *p* = 0.013), trait anger (*r* = 0.275, *p* = 0.016), and lower social QOL (*r* = −0.344, *p* = 0.002). BPD scores were similarly associated with higher state (*r* = 0.242, *p* = 0.031) and trait anger (*r* = 0.291, *p* = 0.011), but had lower QOL in all domains.

**Conclusion:**

In this exploratory analysis, OCPD traits in trauma-exposed Veterans were linked to higher anger and lower social QOL, with effect sizes comparable to BPD traits. This highlights the importance of screening for OCPD traits to inform treatment strategies and improve outcomes, especially given that OCPD traits receive less clinical attention than BPD traits.

## 1. Introduction

Trauma exposure and post-traumatic stress disorder (PTSD) are associated with higher rates of co-occurring personality psychopathology [1,2]. The National Epidemiological Survey on Alcohol and Related Conditions found that both full (OR 2.5–6.7) and partial (OR 1.7–4.1) PTSD was significantly associated with increased likelihood of meeting criteria for all personality disorders (PDs) [1]. Studies examining the intersection between PTSD and PDs in combat Veterans have produced similar findings. For example, a study of 34 combat Veterans in both inpatient and outpatient settings found a PTSD-PD comorbidity rate of 79 %, including borderline (BPD) and obsessive-compulsive personality disorder (OCPD) [2]. With approximately 9.4 % of Veterans diagnosed with PTSD in their lifetime [3], the development of efficacious treatments for this population requires understanding the high rates of co-occurrence between PTSD and PDs, as PDs negatively impact the incidence, symptom severity, and treatment response to PTSD [4,5].

OCPD is the most common PD with an estimated prevalence between 6.5 and 8 % in the general population, 19.9 % among prisoners, and 29.7 % in psychiatric populations [6–8]. OCPD affects approximately 16 % of Veterans [9]. OCPD is an economically costly disorder with high health care utilization rates. One study of 1740 subjects in the Netherlands found that of all PDs, OCPD and BPD were uniquely associated with increased direct and indirect medical costs [10]. Characterized by preoccupation with details, perfectionism, excessive devotion to work and productivity, over-conscientiousness, inability to discard worthless objects, inability to delegate tasks, miserliness, and rigidity and stubbornness [11], OCPD is often underdiagnosed in Veterans as PDs are considered pre-existing conditions that disqualify service members from receiving disability benefits [9]. Because of this, OCPD’s relationship with trauma remains largely understudied in veteran populations. In civilians, trauma exposure is significantly linked to the development of OCPD as evidenced by the high comorbidity between childhood abuse and neglect and OCPD diagnoses [12,13]. Symptoms such as rigidity and socially prescribed perfectionism are theorized to exacerbate the avoidance of recurrent past traumatic experiences, worsening global PTSD symptoms [14]. In addition, perfectionism is thought to be related to avoidance behavior, negative affectivity, and heightened anger in individuals with either OCPD [15,16] or PTSD [17,18], leading to poor social functioning and internal distress.

Importantly, Veterans with obsessive-compulsive traits are more prone to trauma-related anger and increased risk of suicide [17,19]. Anger is a common yet serious problem in Veterans, occurring monthly in approximately 30 % of individuals and disproportionately affecting those diagnosed with PTSD [17]. Complicating matters further, OCPD-associated traits, such as aversion to interpersonal warmth [16] and perfectionism [20] can weaken the therapeutic alliance between Veterans and providers, rendering life-saving treatments less effective [21]. Anger also significantly worsens Veterans’ interpersonal relationships, limiting their social and institutional support [22], which are critical for preventing (1) intimate partner violence and domestic disputes [23], (2) unemployment [24], (3) loneliness and isolation [25], (4) the incidence of physical ailments including coronary heart disease [26], and (5) the risk of physical violence and incarceration [21].

In addition to the impact of OCPD on anger, an OCPD diagnosis is also associated with significant functional impairment [16,27,28] and poor quality of life (QOL) [29]. Although studies have found OCPD to have less of an impact on QOL and functioning than other PDs [30,31], the QOL reduction in individuals with OCPD is still significant and comparable to that of individuals with obsessive-compulsive disorder [28]. While the exact mechanisms through which OCPD impacts QOL are not known, it is plausible that maladaptive levels of perfectionism may lead to missing deadlines, seeking extensions on work or school assignments, and spending excessive amounts of time on otherwise trivial tasks. Others may view individuals with OCPD as controlling and rigid as a result of their difficulty with delegating tasks and tendency to impose their points of view on others. The metacognitive difficulties and emotional dysregulation in OCPD may further contribute to high levels of internalized distress and interpersonal difficulties [16,32,33]. Despite the high prevalence of OCPD in Veterans [2], the impact of OCPD traits on QOL in this population remains understudied.

Importantly, evidence also links OCPD to heightened risk of suicidal ideation, attempts, and non-suicidal self-injury. Though understudied, core OCPD tendencies such as hostility−manifesting as anger, irritability or critical behavior—lead to conflict and rejection, while avoidance limits opportunities for closeness and support. Together, these patterns undermine social connectedness and its protective impact on suicidal risk [34–36]. Unsuppressed anger, in particular, may be a key mechanism linking chronic interpersonal dysfunction in OCPD to elevated suicide risk and in light of the unremitting suicide crisis, merits additional study.

In this exploratory, hypothesis-generating study, we examined the relationship between OCPD traits, state and trait anger, and QOL in a sample of post-911 combat Veterans with moderate to severe problems with anger. Participants were recruited for a separate randomized controlled trial of a cognitive-behavioral therapy for anger in combat Veterans [19]. We include BPD traits as a comparison subgroup within the overall sample given that (1) in these authors’ clinical experience, there is greater emphasis on the diagnosis and treatment of BPD than OCPD, and (2) there is a well-established relationship between BPD traits, maladaptive anger [37] and reduced QOL [38,39].

## 2. Methods

### 2.1. Sample

The sample includes post-911 combat Veterans (*n* = 92) recruited for a randomized controlled trial examining a cognitive behavior treatment for anger problems [19]. This study was conducted between March 2015 and February 2019 and approved by the Providence VA Medical Center Institutional Review Board (IRBnet 1,633,708–11; Clinicaltrials.gov ID NCT02157779). All participants provided informed consent in compliance with the IRB code of ethics by the World Medical Association for Human Subjects. Participants were referred by mental health clinicians from the local VA mental health service or were self-referred in response to brochures or postings. Inclusion criteria included having served in a post-911 deployment, experiencing one or more DSM-5 Criterion A traumatic events while deployed, reporting at least 2 hyperarousal symptoms (irritable behavior/angry outbursts, reckless/self-destructive behavior, hypervigilance, exaggerated startle, problems with concentration, or sleep disturbance), in addition to moderate to severe problems with anger. Participants with a severe substance use disorder, psychotic symptoms, a manic episode within the 3 previous months, current suicidal or homicidal ideation requiring hospitalization, and severe cognitive impairment were excluded from this study. Demographic information was obtained via self-report forms. For a full description of sample characteristics, please refer to Shea et al. 2022 [19].

Basic demographics are outlined in Table 1. Participants were predominantly male (97 %) with an average age of 35.9 years. Most participants identified as White (87 %) and 21 % identified as Hispanic. A little over half (57 %) of participants were married, 63 % were employed, and 59 % had at least some college education. The average number of deployments was 1.61 (*SD 0*.84), and the average number of years since the last deployment was 7.06 (*SD* 3.29). *N* = 44 (48 %) of participants were taking psychotropic medications at the time of assessment.

### 2.2. Assessment of trauma

Subjects were assessed at the initial interview for PTSD using the Clinician Administered PTSD Scale for the DSM-5 (CAPS-5) [40]. Trained interviewers met monthly to discuss ratings of recorded interviews to prevent drift. Exposure to deployment trauma was assessed by CAPS-5 Life Events Checklist.

### 2.3. Assessment of OCPD, BPD, and psychiatric comorbidities

Personality diagnoses and dimensional factors were assessed using the Schedule for nonadaptive and adaptive personality second edition (SNAP-2) [41,42]. The SNAP-2 is a 375-item factor analytically-derived self-report questionnaire that assesses personality traits ranging from the healthy to pathological range. The SNAP-2 consists of 12 primary trait scales and 3 temperament scales that assess 15 personality facets across 3 broad categories: negative affectivity (i.e. neuroticism), positive affectivity (i.e. extroversion), and disinhibition vs constraint (disinhibition and impulsivity vs propriety and workaholism). Personality dimensions include negative temperament, mistrust, manipulativeness, aggression, self-harm, eccentric perceptions, dependency, positive temperament, exhibitionism, disinhibition, impulsivity, propriety, workaholism, entitlement and detachment. The SNAP-2 also includes 13 scales to assess DSM-5 PD diagnoses. The SNAP-2 scales have strong convergent validity (convergent *rs* ranging from 0.4 to 0.7), internal consistency (median alphas between 0.80 and 0.85), and temporal stability for ranges between 7 days and 4 months (0.87) [36]. OCPD and BPD total scores were calculated by summing the items in the SNAP-2 scales that correspond to DSM-5 section 2 criteria for OCPD (Cronbach’s alpha of 0.74) and BPD (Cronbach’s alpha of 0.71) respectively. The Structured Interview for the DSM-5 [43] was used to determine rates of major depressive disorder (MDD), alcohol use disorder (AUD), and substance use disorders (SUD).

### 2.4. Assessment of anger

State and Trait Anger were assessed using the State-Trait Anger Expression Inventory Version 2 (STAXI-2) [44], a 57-item self-report inventory which measures 4 separate scales of anger: 1) the intensity of anger as an emotional state (state anger), 2) the tendency to experience anger as a personality trait (trait anger), 3) anger expression, as measured by both the expression of anger towards other persons or objects in the environment (anger-expression out) vs suppressing anger feelings (anger-expression in) 4) anger control, as measured by the tendency to control angry feelings by preventing anger expression towards persons or objects in the environment (anger-control out), and by controlling angry feelings through internal measures (anger-control in). The STAXI-2 has been validated in both clinical and nonclinical samples, and high test-retest reliability (with the exception of state anger) and internal consistency alpha coefficients all above 0.7 for each scale [45]. In this study, only state and trait anger scales were utilized.

### 2.5. Assessment of QOL

QOL was assessed using the World Health Organization Quality of Life - Brief Version [46]. The WHOQOL-BREF is a 26-item abbreviated version of the WHOQOL-100 which assesses four main domains of functioning and well-being: physical health, psychological health, social relationships, and environmental factors. The WHOQOL-BREF scores correlate highly (0.89 or above) with WHOQOL-100 scores, and this tool demonstrates good content validity, internal consistency, and test-retest reliability [47]. Chronbach’s alpha for the WHOQOL-BREF in this study was 0.622.

### 2.6. Statistical analysis

The number of individuals meeting each OCPD and BPD criterion were calculated using the corresponding SNAP-2 items respectively. We then calculated the number of individuals who met full DSM-5 criteria for OCPD and BPD by adding up the number of criteria satisfied for each participant. The participant must have met at least 4/8 criteria for OCPD and 5/9 criteria for BPD. A chi square test between individuals meeting OCPD criteria and BPD criteria was utilized to determine whether there was a significant relationship between individuals meeting criteria for OCPD and BPD.

In order to understand the impact of OCPD traits and anger, OCPD total continuous scores were calculated for all participants (regardless of whether they met full DSM-5 criteria for either disorder) by adding up the number of corresponding SNAP-2 items met. A Wilks-Shapiro test for normality was significant for the OCPD total continuous score (*p* = 0.045), suggesting a nonparametric distribution. Spearman’s rank correlations were utilized to examine the relationship between the SNAP2 OCPD total continuous score and the STAXI-2 state and trait anger measures. The BPD total continuous score was similarly calculated by adding up the number of BPD items met for each participant, and Spearman’s rank correlations were run for the BPD total continuous score and STAXI-2 state and trait anger measures. Given the relationship between PTSD symptoms and anger, we then conducted a partial Spearman’s rank correlation analysis for the OCPD total continuous score, BPD continuous score and STAXI-2 anger measures while controlling for total CAPS PTSD symptom score (the sum of the number of criteria met; Cronbach’s alpha = 0.38) and the total symptom severity score (the sum of the item severity ratings; Cronbach’s alpha = 0.77). The magnitude of the correlations between OCPD and BPD scores and anger measures were compared both qualitatively and using a Cohen’s Q. Finally, Spearman’s rank correlations were used to examine the relationship between the OCPD total continuous score, BPD total continuous score, and the WHOQOL-BREF QOL domains. Spearman’s Rho effect size cutoffs were based on Rea and Parker, 1992 (small effect: 0.1–0.2; medium effect: 0.2–0.4; relatively strong effect 0.4–0.6; strong effect 0.6–0.8; very strong effect 0.8–1.0) [48].

## 3. Results

### 3.1. Psychiatric diagnoses and hyperarousal symptoms

Rates of MDD, AUD, and SUD are shown in Table 1. As expected in this population of combat-exposed Veterans with hyperarousal symptoms, 65 % of participants met DSM-5 criteria for PTSD. Fifty percent of participants met criteria for MDD and 16 % met criteria for AUD or SUD. Table 1 depicts the percentage of participants who experienced specific hyperarousal symptoms, with the most common symptom being irritability/angry outbursts, followed by hypervigilance.

### 3.2. OCPD, BPD and anger

In this sample of 92 Veterans, 23 participants met full DSM-5 criteria for BPD (25 %) and 17 met full criteria for OCPD (18.5 %). Of the 17 participants who met criteria for OCPD, 6 also met the full criteria for BPD. Results of a chi square test between participants who met OCPD criteria and BPD criteria showed no significant association (*x*^*2*^ = 0.684, *df* = 1, *N* = 79). Table 2 depicts the percentages of all participants who endorsed each OCPD-specific criterion on the SNAP-2.

Table 3 summarizes the Spearman’s correlations between OCPD scores, BPD scores, and anger. The OCPD total continuous score was significantly correlated with both state anger (*r* = 0.276, *p* = 0.013) and trait anger (*r* = 0.275, *p* = 0.016) with moderate effect sizes. This remained significant when controlling for CAPS symptom score (state anger *r* = 0.276, *p* = 0.017; trait anger *r* = 0.253, *p* = 0.029) (Table 4). Although the association with trait anger lost significance when controlling for the CAPS severity score (state anger *r* = 0.236, *p* = 0.043; trait anger *r* = 0.198, *p* = 0.091), the effect size of the correlations remained similar. Of note, the OCPD total continuous score itself was not significantly correlated with either the CAPS-5 severity (*r* = 0.163, *p* = 0.146) or symptom scores (*r* = 0.066, *p* = 0.559). Similarly, BPD total continuous scores were significantly associated with both state anger (*r* = 0.242, *p* = 0.031) and trait anger (*r* = 0.291, *p* = 0.011) with similarly moderate effect sizes. When controlling for CAPS-5 symptom scores, the relationship between the BPD continuous score and both state and trait anger lost significance (state anger *r* = 0.150, *p* = 0.201; trait anger *r* = 0.192, *p* = 0.101). A similar pattern emerged when controlling for CAPS-5 severity scores (state anger *r* = 0.060, *p* = 0.609; trait anger *r* = 0.084, *p* = 0.476). Of note, the BPD total score was significantly associated with both the CAPS-5 severity (*r* = 0.395, *p* ≤0.001) and symptom (*r* = 0.372, *p* = 0.001) scores, which may explain the loss of significance when these variables were added to the model. The Cohen’s Q between correlation coefficients for OCPD and BPD total scores and state anger was −0.036. The Cohen’s Q between correlation coefficients for OCPD and BPD total scores and trait anger was 0.017. These values correspond to small effect sizes, which indicates that the strength of these correlations is not statistically different.

Given the significant correlations between the OCPD total continuous score and state and trait anger, we explored whether specific OCPD criteria had stronger correlations with anger than others. We found that of the 8 OCPD criteria, inflexible morality and ethics was the only criterion correlated with both state (*r* = 0.270, *p* = 0.014) and trait (*r* = 0.236, *p* = 0.035) anger with moderate effect sizes. Both reluctance to delegate tasks (*r* = 0.256, *p* = 0.024) and rigidity/stubbornness (*r* = 0.414, *p* < 0.001) were associated with trait anger only with moderate and relatively strong effect sizes respectively.

### 3.3. OCPD, BPD, and QOL

The OCPD total continuous score was significantly correlated with lower scores on the social domain of the WHOQOL-BREF (*r* = −0.344, *p* = 0.002) with a moderate effect size, but not the physical (*r* = 0.047, *p* = 0.674), psychological (*r* = −0.57, *p* = 0.614), or environmental (*r* = 0.076, *p* = 0.502) domains. In contrast, the BPD total continuous scores were significantly correlated with lower scores in all QOL domains (physical: *r* = −0.329, *p* = 0.003; psychological: *r* = −0.479, *p* < 0.001; social: *r* = −0.388, *p* < 0.001; environmental: *r* = −0.342, *p* = 0.002) with moderate to relatively strong effect sizes (Table 3). When controlling for CAPS-5 symptom scores (Table 4), these results remained effectively the same. When controlling for CAPS-5 severity scores (Table 5), the results remained largely the same with the exception that the relationship between BPD scores and the physical QOL domain lost statistical significance (*r* = 1, *p* = 0.084), although the effect size remained similar.

## 4. Discussion

In this hypothesis-generating study, we demonstrate that in a sample of trauma-exposed Veterans with problematic anger, OCPD traits are associated with higher levels of anger and lower QOL within the social domain. The OCPD total scores on the SNAP-2 were positively correlated with both state and trait anger with moderate effect sizes, meaning that individuals with OCPD traits are more likely to experience anger more intensely and as a personality trait. These correlations remained significant when controlling for PTSD symptom scores, but only state anger remained significant when controlling for PTSD severity scores. PTSD severity may be an important confounder given the high rates of comorbid PTSD in this population and the strong relationship between PTSD and anger. The OCPD total continuous score alone was not significantly correlated with either the PTSD symptom or severity scores, suggesting they are independent constructs.

These findings are consistent with studies in other populations that have found correlations between a diagnosis of OCPD and significantly higher levels of negative affectivity, trait anger, emotional intensity, and emotional regulation difficulties [15,33,49]. Individuals with OCPD traits hold harsh rules and standards, and violating these rules may result in either inwardly or externally-directed anger [49]. In fact, it is thought that OCPD follows a two-factor model such that individuals may be either more *anxious* and perfectionistic or more *controlling* and rigid [32,50,51]. Those on the anxious side of the spectrum tend to internalize and are more prone to depression and suicidality; like in avoidant PD, synergy between negative affectivity and social isolation are potentially key to the phenotype [52] and heightened suicide risk [53]. Those on the controlling side, on the other hand, tend to externalize and are more prone to anger and aggression [50,51]. The need for interpersonal control in individuals with controlling-dominant OCPD may lead to hostility and outbursts of anger both at home and in the workplace [54]. These theories are consistent with our findings that of the 8 OCPD criteria, only inflexible morality and ethics, reluctance to delegate, and rigidity/stubbornness were associated with the STAXI-2 anger measures. It is also interesting to note that in our sample of Veterans who were pre-selected for having high levels of anger, 65.8 % of participants endorsed the rigidity and stubbornness OCPD criterion. Furthermore, the effect size of the correlation between the rigidity and stubbornness criterion and trait anger was relatively strong, whereas most other correlations only had moderate effect sizes. This may indicate that rigidity and stubbornness represent a transdiagnostic feature of problematic anger.

BPD was selected as a comparison group given that it is well-documented that individuals with BPD struggle with problematic anger and often have significant interpersonal difficulties and distress [37]. In the authors’ experience, clinicians also tend to pay more attention to the diagnosis and treatment of BPD than OCPD, and the relationship between trauma and BPD has been well-studied [55,56] in contrast to OCPD. Unsurprisingly, BPD traits were also significantly associated with both state and trait anger. However, it was notable that the differences in magnitude of the correlation coefficients between OCPD traits and state/trait anger and BPD traits and state/trait anger were small, both qualitatively and using Cohen’s Q. This suggests that while OCPD is often overlooked clinically, it may have as strong of an impact on pathologic anger in this population and represents a critical gap in care. Although these findings are preliminary and require validation, we believe they represent an important signal that OCPD traits may be playing a larger role in pathologic anger than previously suspected in Veterans.

Despite the need for validation, the results of this pilot study have significant implications for clinical practice. Anger in Veterans has been linked to adverse functional outcomes such as intimate partner violence and domestic disputes, unemployment [24], loneliness and isolation [25]. Higher levels of problematic anger also have been associated with poorer responses to PTSD treatment [57] and is thought to be a mediator between PTSD and increased suicidal ideation and risk of completed suicide [22]. For example, in a sample of 545 community dwelling Veterans, PTSD was indirectly related to increased suicide risk via both depressive symptoms and internally-directed anger [58]. In a different longitudinal study of 319 post-9/11 Veterans followed over the course of one year, changes in anger over time were found to mediate the dynamic relationship between suicide risk and PTSD severity [59]. This is compounded by the fact that OCPD itself is associated with an increased lifetime risk of suicidal ideation and suicide attempts [60]. These findings highlight the importance of anger as a transdiagnostic symptom that has significant implications for suicide risk, especially as Veterans’ age and sex adjusted risk of suicide is already 71.8 % higher than the general population [61].

Upon examining OCPD and QOL in our exploratory analysis, we found that OCPD traits were not significantly associated with physical, psychological, or environmental impairment in QOL, but were significantly associated with impairment in social QOL with a moderate effect size. This contrasts with BPD traits, which were significantly associated with impairment in all four QOL domains with moderate to relatively strong effect sizes. Although not statistically significant, the correlation between OCPD and psychological QOL (*r* = −0.57) was slightly stronger than for BPD (*r* = −0.479), suggesting a relatively strong effect that may reach significance with greater power.

The impairment in the social QOL domain in OCPD is consistent with prior studies demonstrating high rates of hostile-dominant interpersonal problems and interpersonal distress in OCPD, as well as reports of cold, vindictive and domineering interpersonal styles and low levels of perspective taking as compared to healthy controls [16]. This is also unsurprising given the high percentage of individuals endorsing rigidity and stubbornness and our finding that OCPD traits may be associated with higher levels of state and trait anger, which may contribute to interpersonal difficulties [22]. This finding may be particularly important in a population with already high rates of both psychiatric conditions that negatively impact QOL such as PTSD [62], other PDs [63], and MDD [64].

Importantly, identifying OCPD in Veteran populations may help guide treatment. While there are few clinical trials of selective-serotonin reuptake inhibitors (SSRIs) in OCPD, a small randomized clinical trial in which 24 participants with OCPD were randomized to receive either fluvoxamine (50–100 mg/day) or placebo for 12 weeks found a significant improvement in symptoms in the active treatment group [65]. In another small study of 308 patients with MDD and comorbid PDs, there was some evidence that citalopram was more effective than sertraline for individuals with comorbid OCPD [66]. In terms of psychotherapeutic interventions, there is evidence that individuals with OCPD and problematic perfectionism can benefit from both cognitive-behavioral therapy and acceptance and commitment therapy [32]. Metacognitive interpersonal therapy, an integrative approach that combines aspects of psychodynamic, mentalization, and narrative-based therapies [33] may also help individuals with OCPD better understand mental states in themselves and others. This type of therapy directly addresses perfectionism as a coping mechanism and helps individuals with OCPD reconnect with aspects of the self that are unrelated to perfectionism. This may help individuals recognize how past experiences may be linked to thoughts, feelings, and behaviors. For instance, OCPD is often associated with a pervasive sense of inadequacy and sensitivity to criticism. Metacognitive approaches may help patients understand that their interpersonal reactions to perceived criticism are not predetermined, but rather a consequence of past experiences and subsequent automatic thought patterns [67]. Several case series have demonstrated symptom remission of OCPD with metacognitive therapy, making this a promising avenue for further exploration [67,68].

This study has several limitations that should be considered. First, it comprises an exploratory analysis of data collected for a parent study examining a cognitive behavior treatment for anger problems. This study was not designed for assessing OCPD traits. As such, our results should be considered hypothesis-generating and not hypothesis-testing; findings should be confirmed with future purpose-designed studies. While there is precedent for adding SNAP-2 items as a proxy for measuring PD severity [69], the SNAP-2 does not directly assess PD severity. A continuous measure with a larger number of items and more variability such as the Pathological Obsessive Compulsive Personality Scale [70] would be preferable. Understanding how overall PD severity impacts the relationship between OCPD and anger is an important area of exploration, particularly given the large overlap in clinical characteristics between different PDs. Finally, we note that the cross-sectional design of this study precludes our ability to detect temporal relationships between OCPD traits and anger, and the predominantly White male population limits the generalizability of our preliminary findings.

Due to the exploratory nature of this study, we did not correct for multiple comparisons. This decision was made partially due to power considerations. Our sample was already selected for having moderate to severe problems with anger. Thus, differences between anger measures in participants with and without OCPD traits may be less than in a general veteran population and thus more difficult to detect. Despite these limitations, these results emphasize the importance of future purpose-driven studies of OCPD traits and their impact anger, QOL, and treatment outcomes in trauma-exposed Veterans. Furthermore, understanding whether the relationship between OCPD and anger are stronger in the controlling vs anxious phenotypes may provide insights into transdiagnostic traits contributing to problematic anger in Veterans, leading to improved treatment paradigms and clinical outcomes.

## 5. Conclusion

In a sample of trauma-exposed Veterans with problematic anger, OCPD traits are associated with higher levels of anger and lower QOL. The magnitude of correlations of OCPD traits on anger and social QOL are comparable to that of BPD traits, highlighting the importance in this population of identifying and treating OCPD – a diagnosis that is often overlooked. These findings are consistent with previous studies linking OCPD to both problematic anger and interpersonal difficulties. This exploratory study highlights the importance of identifying and treating OCPD traits in combat-exposed Veterans, particularly given the implications of problematic anger to interpersonal functioning and suicide risk. Further work is needed to understand the longitudinal relationships between OCPD traits and anger, QOL, and treatment outcomes in this population.

## Figures and Tables

**Table 1:** Sample characteristics.

	Participants *N* = 92
Demographics	
Age, *M* (*SD*)	35.9 (9.4)
Sex (% male)	89 (97)
Race/ethnicity, *n* (%)	
Black or African American	6 (7)
White	80 (87)
More than one race	5 (5)
other	12 (13)
Ethnicity, *n* (%) Hispanic	19 (21)
Married/living together, *n* (%)	52(57)
High school or less, *n* (%)	22(24)
Some College, *n* (%)	54(59)
College degree or more, *n* (%)	16 (17)
Employed, *n* (%)	58 (63)
Psychiatric Diagnoses	
Current PTSD, *n* (%)	60 (65)
Current MDD, *n* (%)	46 (50)
Current AUD, *n* (%)	15 (16)
Current SUD, *n* (%)	15 (16)
Hyperarousal Symptoms, *n* (%)	
Irritable behavior/angry outburst	89 (96.6)
Reckless/Self-destructive behavior	25 (27.2)
Hypervigilance	84 (91.3)
Exaggerated Startle	64 (69.6)
Problems with concentration	72 (78.2)
Sleep disturbance	76 (82.6)

Abbreviations: PTSD = post-traumatic stress disorder; MDD = major depressive disorder; AUD = alcohol use disorder; SUD = substance use disorder.

**Table 2:** Percentage of participants meeting each OCPD criterion.

	Participants Meeting Criterion, n (%) *N* =*92*
OCPD Criterion	
Preoccupation with details, rules, schedules	14/84 (17.7)
Perfectionism that interferes with task completion	20/83 (24.1)
Excessive devotion to work	18/84 (21.4)
Overconscientious, scrupulous, inflexible morality	27/84 (32.1)
Unable to discard worn-out or worthless objects	26/83 (31.3)
Reluctant to delegate tasks/work with others	20/82 (24.4)
Miserly spending style towards self and others	11/84 (13.1)
Rigidity and stubbornness	54/82 (65.8 %)

Abbreviations: OCPD = obsessive-compulsive personality disorder.

**Table 3:** Spearman rank correlation coefficients for OCPD and BPD total continuous scores and anger measures.

	OCPD total continuous score	BPD total continuous score	State Anger	Trait Anger	Physical QOL	Psychological QOL	Social QOL	Environmental QOL
OCPD total continuous score	x	0.157	0.276*	0.275*	0.047	−0.57	−0.344**	0.076
BPD total continuous score		x	0.242*	0.291*	−0.329**	−0.479***	−0.388***	−0.342**
State Anger			x	0.507**	−0.171	−0.272**	−0.280**	0.018
Trait Anger				x	−0.137	−0.293**	−0.321**	0.046
Physical QOL					x	0.601**	0.292**	0.402**
Psychological QOL						x	0.570**	0.506**
Social QOL							x	0.380**
Environmental QOL								x

Abbreviations: OCPD = obsessive-compulsive personality disorder; BPD = borderline personality disorder; QOL = quality of life.

* significant at the 0.5 level.

** significant at the 0.01 level.

*** significant at the 0.001 level.

**Table 4:** Partial Spearman rank correlation coefficients for OCPD and BPD total continuous score, anger measures, and QOL while controlling for CAPS symptom scores.

	OCPD total continuous score	BPD total continuous score	State Anger	Trait Anger	Physical QOL	Psychological QOL	Social QOL	Environmental QOL
OCPD total continuous score	x	0.114	0.276*	0.253*	0.071	−0.24	−0.36**	0.105
BPD total continuous score		x	0.150	0.192	−0.251*	−0.362**	−0.334**	−0.364**
State Anger			x	0.424**	−0.186	−0.166	−0.180	0.131
Trait Anger				x	−0.85	−0.220	−0.269	0.164
Physical QOL					x	0.582**	0.226	0.404**
Psychological QOL						x	0.226	0.404**
Social QOL							x	0.457**
Environmental QOL								x

Abbreviations: OCPD = obsessive-compulsive personality disorder; BPD = borderline personality disorder; CAPS = Clinician Administered PTSD Scale for the DSM-5; QOL = quality of life.

* significant at the 0.5 level.

** significant at the 0.01 level.

**Table 5:** Partial Spearman rank correlation coefficients for OCPD and BPD total continuous score, anger measures, and QOL while controlling for CAPS severity scores.

	OCPD total continuous score	BPD total continuous score	State Anger	Trait Anger	Physical QOL	Psychological QOL	Social QOL	Environmental QOL
OCPD total continuous score	x	0.063	0.236*	0.198	0.128	−0.066	−0.320**	0.103
BPD total continuous score		x	0.060	0.084	−0.202	−0.315**	−0.260*	−0.409**
State Anger			x	0.322**	−0.101	−0.13	−0.51	0.126
Trait Anger				x	0.050	−0.19	−0.99	0.157
Physical QOL					x	0.535**	0.132	0.439**
Psychological QOL						x	0.376**	0.548**
Social QOL							x	0.380**
Environmental QOL								x

Abbreviations: OCPD = obsessive-compulsive personality disorder; BPD = borderline personality disorder; CAPS = Clinician Administered PTSD Scale for the DSM-5; QOL = quality of life.

* significant at the 0.5 level.

** significant at the 0.01 level.

## Data Availability

The data that support the findings of this study may be available upon reasonable request to the corresponding author following all relevant regulations.

## Acronyms:

PTSD: post-traumatic stress disorder
PD: personality disorder
BPD: borderline personality disorder
OCPD: obsessive-compulsive personality disorder
